# Experience using artificial intelligence in the digital transformation of education: benefits and challenges

**DOI:** 10.3389/frai.2026.1756665

**Published:** 2026-04-20

**Authors:** Arturs Medveckis, Tamara Pigozne, Rita Birzina, Ivita Pelnena

**Affiliations:** 1 Riga Technical University Liepaja Academy, Centre for Management and Social Sciences, Liepaja, Latvia; 2 Faculty of Educational Sciences and Psychology, University of Latvia, Rīga, Latvia; 3 Faculty of Medicine and Life Sciences, University of Latvia, Rīga, Latvia; 4 Riga Technical University Liepaja Academy, Centre for Humanities and Arts, Liepaja, Latvia

**Keywords:** adult educators, adult learning, attitude towards the use of artificial intelligence, challenges related to the use of artificial intelligence, the impact of using artificial intelligence

## Abstract

**Aim:**

The aim of the research is to analyse teachers and adult educators’ experiences of the application of artificial intelligence in the digital transformation of education.

**Methods:**

In the study the quantitative data collection method—a questionnaire—“International Survey on Artificial Intelligence in Higher Education, Training and Adult Learning” and data processing methods for secondary data collection—descriptive statistics (Mean, Median, Mode, Standard Deviation), and the Mann–Whitney test to determine the statistical significance of differences between two independent target groups (teachers and adult educators) have been applied.

**Results:**

The research sample consisted of representatives of educational institutions of Latvia—34 teachers and 83 adult educators. The descriptive statistics and Mann–Whitney test results show that both teachers and adult educators similarly assess the impact of AI application on performance and work efficiency and productivity, decision-making, problem-solving, awareness formation and interdisciplinary concept application skills, mental health, learning outcomes and challenges related to AI use (*p* ≥ 0.05). Teachers, compared to adult educators, have a higher opinion of the application of artificial intelligence for work purposes. Adult educators have a higher opinion of the impact of AI on the development of learners’ awareness formation skills, learners’ employment and their work performance, and physical and social health (*p* ≤ 0.05).

**Conclusion:**

AI is a new global reality that opens up new paths for knowledge acquisition, whereas the social environment is also facing new challenges. AI tools can be used by a wide range of users who have prior knowledge and skills in constantly changing IT application. Despite the inertia of the education system and the length of bureaucratized decision-making, proactive action is needed that would balance the technological development with the acquisition of new knowledge and skills based on high moral standards at all levels of education, involving high-tech implementers and cooperating with educational staff, scientists and other social partners. A descriptive cross-sectional study design has been chosen for the study and the instrument applied is the survey “International Survey on Artificial Intelligence in Higher Education, Training and Adult Learning” developed by the Singapore Institute of Adult Education within the framework of the 3rd network “Professionalization of Adult Educators in ASEM Countries” of the Asia-Europe Lifelong Learning and Education Research (ASEM LLL Hub).

## Introduction

Artificial intelligence in the 21st century has become a transformative force, tool and resource that can be used to advance various sectors, including education (Gorriz et al., 2020), especially in educational contexts such as performance and learning outcomes, work purposes, productivity and efficiency, development of students’ skills, health, challenges. Although the results of meta-analytic studies show that research on artificial intelligence has been conducted in six of the world’s seven continents and education is one of the sectors where it is most widely used (Crompton and Burke, 2023), at the same time, scientists emphasize the need to continue the research with an aim to analyse the possibilities and risks of application of artificial intelligence in the context of education, including in Latvia (Hrastinski et al., 2019; Zawacki-Richter et al., 2019). The most commonly cited risks are the need to address ethical issues, the risks of plagiarism and the lack of transparency in the application of artificial intelligence (Crawfold et al., 2023; Lund et al., 2023).

The results of systematic review show—alignment of artificial intelligence with educational goals and effective addressing of the related problems are cited as essential factors in a positive attitude and perception promotion (Matos et al., 2025). These findings highlight the need for comprehensive implementation strategies that include technical, ethical, social and educational considerations. Studies highlight the value of AI not only in the enrichment of teaching and learning experiences, but also in the improvement of resource availability, rationalization of administrative processes and encouragement of innovation in education (Al-Zahrani and Alasmani, 2024).

Each level of education and field has its own priorities and resources for AI application. As a result of the experience gathered by several scientists, it has been found that the application of AI in engineering education includes not only the field of new technologies and resources for specific engineering problem solution, but also the application of smart technologies and resources to support and improve the learning process (Liu et al., 2025). Preparation of future teachers for STEM programmes plays a crucial role, where the importance can be seen in the future educators’ enthusiasm and willingness to learn and use various artificial intelligence tools in order to improve the quality of education, customize the learning experience and promote student achievement and engagement (Sun et al., 2024).

AI is impacting higher education, bringing challenges, risks and opportunities for both university academic staff and students. Considering the particularly broad implementation of generative AI in higher education, the experience of academic staff is important in data collection, presenting a more profound understanding and providing a personalized support, respecting the students’ needs (Doģan et al., 2024).

Artificial intelligence applications are most relevant in adult education (Peng, 2024) as they offer potentially transformative ways to design and implement personalized and adaptive learning environments and training systems (Oberdieck and Moch, 2024), marking the transition from fixed curricula to adaptive learning experiences, emphasizing education as a continuous journey rather than a static destination, and it is the application of artificial intelligence that is a catalyst for change, opening up opportunities to direct one’s own learning that is aligned with personal interests and needs (Fidalgo and Thormann, 2024).

AI has a dual impact on employment, offering opportunities and creating challenges: on the one hand, job losses as AI takes over routine tasks in manufacturing, retail and customer service; on the other hand, retraining and upskilling of the workforce, essential for AI-led workplace transformation, is essential to address skills shortages and improve adaptability (Patil, 2024).

In order to implement the research objective—to analyse the experience of the application of artificial intelligence in the digital transformation of education—the following research questions have been raised:

*How many teachers and adult educators use artificial intelligence for their work?*

*How do respondents assess the impact of artificial intelligence on performance, work productivity and efficiency?*

*What is the respondents’ attitude towards the application of artificial intelligence for work purposes?*

*How do respondents assess the impact of the application of artificial intelligence on* var*ious aspects of education?*

*What are the long-term impacts of the application of artificial intelligence in an educational context?*

*What are the challenges associated with the application of artificial intelligence in an educational context?*

*What is the statistical significance of the differences depending on the respondents’ profile in the assessment of the impact of the application of artificial intelligence and attitude towards its use, as well as the challenges associated with it?*

## Methodology

A descriptive cross-sectional study design has been chosen for the study and the instrument applied is the survey “International Survey on Artificial Intelligence in Higher Education, Training and Adult Learning” developed by the Singapore Institute of Adult Education within the framework of the 3rd network “Professionalization of Adult Educators in ASEM Countries” of the Asia-Europe Lifelong Learning and Education Research (ASEM LLL Hub).

Respondents’ answers to closed-ended questions about the impact of the application of artificial intelligence on student performance, work productivity and efficiency, skill development, work performance, health and learning outcomes, attitude towards the use of artificial intelligence for work purposes, as well as challenges related to the use of artificial intelligence in the educational context have been rated on a 5-point Likert scale (1 = strongly disagree, 2 = disagree, 3 = neither agree nor disagree, 4 = agree, 5 = strongly agree). One hundred and seventeen respondents have taken part in the study – 34 teachers and 83 adult educators from various educational institutions in Latvia, where general basic education, secondary and higher education programmes, as well as professional continuing education and professional development education programmes are implemented. Respondents were reached using a publicly available database of educational institutions, sending questionnaires to potential respondents in the GoogleDocs environment; the survey was anonymous and voluntary.

Cronbach’s alpha coefficient for test reliability (*α* = 0.83) indicates good internal consistency. Descriptive statistics has been used in the first stage of data processing, calculating the arithmetic mean, median, mode and standard deviation.

Since the results of the Kolmogorov–Smirnov test for the determination of the empirical distribution show that it does not correspond to the normal distribution (*p* > 0.05), which determines the use of non-parametric methods, the Mann–Whitney test has been used to determine statistical significance depending on the respondents’ profile in the second stage of data processing.

A positive decision on the compliance of the study with the principles of research ethics, risk–benefit analysis and compliance with the requirements for the protection of the rights of research participants was made at the meeting of the Humanities and Social Sciences Research Ethics Committee of the University of Latvia on June 12, 2024.

## Results and discussion

Recent scientific findings position teachers as active participants in shaping the educational role of artificial intelligence, analyzing the complex navigation of pedagogical and strategic choices in the educational context (Tripathi et al., 2025). Of the respondents involved, 81.0% of teachers and 88.8% of adult educators use artificial intelligence. The large proportion of users in Latvia is associated with a wide range of support mechanisms (platforms, continuing education courses, etc.) (see Table 1).

**Table 1:** The impact of application of artificial intelligence on performance, work productivity and efficiency.

Profile	Mean	Median	Mode	SD	*p*
Using AI improves my performance in my work					
Teachers	3.53	4	4	0.84	*p* > 0.05
Educators	3.63	4	4	0.95	
Using AI increases the productivity in my work					
Teachers	4.06	4	4	0.74	*p* > 0.05
Educators	3.78	4	4	0.98	
Using AI enhances my effectiveness (doing the right things) in my work					
Teachers	3.71	4	4	0.98	*p* > 0.05
Educators	3.62	4	4	0.95	

*p < 0.05; **p ≤ 0.01; ***p ≤ 0.001.

Both teachers and adult educators believe that the use of artificial intelligence improves their performance at work, increases work productivity and efficiency. Although no statistically significant differences have been found in the assessment of the impact of the use of AI on performance, work productivity and efficiency (*p* > 0.05), the results of descriptive statistical analysis show that teachers value the increase in work productivity (Mean = 4.06; Me = 4; Mo = 4) and efficiency (Mean = 3.71; Me = 4; Mo = 4) more, while adult educators believe that the use of artificial intelligence improves their performance (Mean = 3.63; Me = 4; Mo = 4).

The results of the study on the impact of AI intelligence tools on teacher productivity and effectiveness are similar to the results of systematic reviews and other studies. As teachers and adult educators have so far devoted a large part of their time to labour-intensive routine work, searching for information, compiling it, systematizing it, etc., then AI significantly reduces the workload of educators in assessment, lesson planning and management, as well as in other areas. By automating and optimizing routine administrative tasks, AI allows educators to devote more time to research and curriculum development and improvement, thus opening up the opportunity to focus on higher-value activities. Overall, scientific research confirms the transformative potential of AI in education, highlighting its ability to streamline operations, increase teaching effectiveness and improve the teachers’ overall well-being. The integration of AI tools into the academic environment offers a new perspective for the optimization of teacher resources, promoting more effective and sustainable teaching practices (Gupta et al., 2024; Sánchez, 2024).

The obtained results of the study also do not contradict the results of other studies, which have concluded that the integration of AI tools improves the performance of both teachers and students. When analysing the performance, the studies mainly analyse assessment tools and their possibilities of application (Sánchez, 2024) (see Table 2).

**Table 2:** Attitude towards the application of artificial intelligence for work purposes.

Profile	Mean	Median	Mode	SD	*p*
I like the idea of using AI in my work					
Teachers	4.24	4	4	0.83	*p* > 0.05
Educators	3.84	4	4	0.89	
I generally have a favourable attitude towards using AI in my work					
Teachers	4.35	4	4	0.78	*p* = 0.008**
Educators	3.80	4	4	0.91	
I believe it is a good idea to use AI in my work					
Teachers	4.29	4	4	0.98	*p* = 0.030*
Educators	3.95	4	4	0.83	

*p < 0.05; **p ≤ 0.01; ***p ≤ 0.001.

Since teachers’ self-efficacy largely depends on digital skills, it is also influenced by the attitude towards the use of AI tools: teachers with a positive attitude towards AI have higher self-efficacy, while educators with a negative attitude have lower self-efficacy (Heine and König, 2025).

There is a statistically significant difference (*p* = 0.008) depending on the respondents’ profile in the assessment of their attitude towards the use of AI at work: teachers rate it higher (Mean Rank = 63.18) compared to adult educators (Mean Rank = 45.34), as well as a statistically significant difference (*p* = > 0.030) in the opinion that the use of artificial intelligence at work is a good idea: teachers agree more (Mean Rank = 60.35) compared to adult educators (Mean Rank = 45.95), who agree less.

Depending on the specifics of the work, the practice of using AI differs for different groups of respondents. The reproductive knowledge transfer is more pronounced in the school environment, AI makes teachers’ daily work easier, so it is considered a good idea, which is the basis for a positive attitude towards the use of AI for work purposes. On the other hand, the interpretive approach is more complex and requires greater creativity, which remains a fundamental and indispensable element in scientific progress and the creation of innovations (Branda et al., 2025) (see Table 3).

**Table 3:** The impact of application of artificial intelligence on the development of students’ skills.

Profile	Mean	Median	Mode	SD	*p*
The development of learners’ creative thinking skills (considers and connects multiple ideas and information to form solutions or develop new ways of working.)					
Teachers	1.57	1	1	0.87	*p* > 0.05
Educators	1.95	2	1	0.82	
The development of learners’ decision-making skills (Involves the process of implementing a structured decision or course of action from multiple sources of information)					
Teachers	1.76	2	1	0.83	*p* > 0.05
Educators	2.10	2	2	0.78	
The development of learners’ problem-solving skills (refers to the ability to handle difficult or unexpected situations by generating effective and efficient solutions)					
Teachers	1.52	1	1	0.75	*p* = 0.005**
Educators	2.09	2	2	0.79	
The development of learners’ sense making skills (interprets and analyses information to identify or recognise patterns and opportunities)					
Teachers	1.67	1	1	0.79	*p* > 0.05
Educators	2.00	2	2	0.79	
The development of learners’ transdisciplinary thinking skills (applies concepts from multiple disciplines to supplement their knowledge to make decisions and solve problems.)					
Teachers	1.90	2	1	0.88	*p* > 0.05
Educators	2.26	2	3	0.75	

*p < 0.05; **p ≤ 0.01; ***p ≤ 0.001.

A statistically significant difference (*p* = 0.005) exists in the assessment of problem-solving skills as a result of the use of artificial intelligence: the adult educators’ assessment is higher (Mean Rank = 54.98) compared to the teachers’ assessment (Mean Rank = 35.86). The results can be explained by the fact that teachers are more focused on improving specific skills, while adult educators offer to search for and find complex solutions.

Adult educators have a stronger ability to combine multiple ideas in order to find innovative solutions. Making reasoned decisions is based on logic and critical analysis of facts, which explains the presence of deeper understanding skills, analysing information and identifying already tested models. One-sided selection of information creates a breeding ground for populism, which is why the development of creative and interdisciplinary thinking skills and a holistic approach are important. AI provides students with immediate access to problem-solving tools, and these tools encourage students to explore alternative solutions that can promote critical thinking without relying on primarily generated answers. However, there are still concerns about the impact of AI on critical thinking (Gerlich, 2025), as the skills of AI tools application and their continuous improvement do not eliminate the need for intellectual effort. Sometimes AI is a way to consider new alternatives, but not the final conclusion; it still remains up to the researcher (see Table 4).

**Table 4:** The impact of the application of artificial intelligence on student performance, health, and learning outcomes.

Profile	Mean	Median	Mode	SD	*p*
Learners’ employability					
Teachers	1.86	2	2	0.47	*p* = 0.000***
Educators	2.41	2	2	0.58	
Learner’s work performance					
Teachers	2.05	2	2	0.74	*p* = 0.011
Educators	2.49	3	3	0.57	
Learners’ mental health (stress, anxiety, etc.)					
Teachers	1.81	2	2	0.68	*p* > 0.05
Educators	2.08	2	2	0.68	
Learners’ physical health					
Teachers	1.62	2	2	0.59	*p* = 0.016*
Educators	1.95	2	2	0.54	
Learners’ social health (ability to interact and form meaningful relationships with others)					
Teachers	1.38	1	1	0.59	*p* = 0.006***
Educators	1.81	2	2	0.65	
Learning outcome					
Teachers	2.05	2	2	0.59	*p* > 0.05
Educators	2.28	2	3	0.72	

*p < 0.05; **p ≤ 0.01; ***p ≤ 0.001.

A statistically significant difference (*p* = 0.000) exists in the assessment of the impact of the use of artificial intelligence on student employment: adult educators assess the impact of the use of artificial intelligence on student employment higher (Mean Rank = 55.93) compared to teachers (Mean Rank = 32.21).

Students’ work performance has a higher added value if more time is devoted to analytical work, expressions of creativity, based on information obtained in a short time and the proposed models, the creative and interpretative use of which opens up new perspectives for innovations—the development and implementation of new products, which are essential in competitive conditions, and opens up prospects for employment.

In turn, there is a statistically significant difference in the assessment of the impact of the use of artificial intelligence on students’ social (*p* = 0.006) and physical health (*p* = 0.016), as well as work performance (*p* = 0.011): adult educators rate it higher (Mean Rank = 54.73; Mean Rank = 54.00; Mean Rank = 54.42), compared to teachers’ assessment (Mean Rank = 36.79; Mean Rank = 39.57; Mean Rank = 37.98).

The ever-increasing amount of time we spend in the digital environment today has an impact on everyone’s health, which is why not only an ergonomic environment, but also certain work hygiene is necessary. The information content is based on certain algorithms that can divert IT users from the originally defined cognitive goal, manipulating the AI user, often affecting psycho-emotional balance and reinforcing addictive motivational mechanisms (see Table 5).

**Table 5:** Challenges related to the application of artificial intelligence in the educational context.

Profile	Mean	Median	Mode	SD	p
Data security and privacy (e.g., collection of personal information)					
Teachers	3.38	3	2	1.24	p > 0.05
Educators	3.61	4	5	1.34	
Bias and discrimination (e.g., bias in AI algorithms)					
Teachers	2.95	3	2	1.43	p > 0.05
Educators	3.54	4	5	1.37	
Transparency and explainability (e.g., decision making using AI)					
Teachers	3.52	4	5	1.32	p > 0.05
Educators	3.73	4	4	1.21	
Job displacement and economic impact (e.g., job function being replaced by AI)					
Teachers	2.62	2	2	1.59	p > 0.05
Educators	2.78	3	3	1.37	
Accountability and responsibility (e.g., over reliance on AI)					
Teachers	3.90	4	5	1.26	p > 0.05
Educators	3.75	4	5	1.17	

*p < 0.05; **p ≤ 0.01; ***p ≤ 0.001.

In the Systematic Review, the biggest challenges for using AI in the digital transformation of education are technical barriers, management support, employees’ distrust of artificial intelligence and resistance to changes, transparency and explainability, responsible use of predictive analytics, data governance and intellectual property (Kavak and Rusu, 2025).

There are no statistically significant differences in the assessment of challenges related to the use of artificial intelligence depending on the profile of the respondents. This means that both teachers and adult educators see the biggest challenges related to accountability and responsibility, transparency and explainability, as well as data security and privacy. The humanities and social sciences are characterized by a high data flow of sensitive information, which raises concerns about privacy. The hard sciences also face a reserved attitude when sharing intellectual property, as there is a possibility that authorship may not be respected in the public environment. Employers are not immune to the AI offer when choosing the criteria for the personnel selection, which may be based on stereotypes about professional suitability. By delegating the performance of routine functions to AI, concerns arise about the desire of educational process managers and administrators to review the compliance of the number of students with the salary rate of one educator, if it is not taken into account that the reduction in the time spent performing routine functions opens up the opportunity for deeper content, methodological improvement and research-based studies.

Making any administrative decisions, determination of the quality of staff creative work or knowledge transfer and quality criteria, as well as performance acceptance, depends on the personal responsibility of everyone involved in the processes, where AI tools and offered solutions have a recommendatory function.

## Conclusion

In the educational process, AI is already a crucial resource that affects performance, work productivity and effectiveness. Effectiveness and productivity depend on the quality of implementation, contextual relevance and professional development support, whereas the relationship between effectiveness, productivity and educational quality must be balanced so that labour resource saving does not jeopardize deeper learning processes and improves, rather than diminishes, the quality of education in the long term (Ehtsham et al., 2025). The performance has mainly been analysed in relation to assessment applying AI tools, emphasizing objectivity, reliability, accuracy and transparency, predicting improvements in educational quality in the long term (Almubrak et al., 2024).

As the paradigm shift from AI as a substitute for teacher tasks to the latest research-based insights into teacher enhancement and intelligent partnership (Ehtsham et al., 2025) has taken place, the attitude towards the application of artificial intelligence for work purposes by teachers and adult educators are primarily influenced by the adaptive capabilities of current digital solutions.

Both teachers and adult educators have mastered the general skills of the application of AI intelligence. However, adult educators have higher skills in rationally evaluating information, synthesizing and creatively developing innovative solutions based on knowledge and personal experience. Problem-solving abilities improve when AI offers alternative solutions that the learner can adapt to an unexpected situation in order to find a positive solution, which also affects the ability to recognize patterns and see their connections with other areas of science.

The impact of the application of artificial intelligence on student performance is higher in the opinion of adult educators than in the opinion of teachers, which can be explained by the earlier involvement and readiness of older learners to compete in the labour market. Mental and physical health affects learning outcomes, which is felt more acutely by adult educators who have identified physical and psychological strain in real-life interactions with students, is based on greater responsibility and performance expectations from learners. Both physical, mental and social health can be affected if ergonomics and occupational hygiene are not sufficiently observed; the ability to manage work and rest regimes must be maintained, where social health must be maintained through direct communication, not replacing it with the application of digital platforms or virtual reality.

The rapid entry of AI into the social environment and especially into the education system should be perceived as a challenge of a new paradigm, which has a broader resonance in the violation of traditional ethical boundaries, both as a deliberate destructive action and an unconscious action due to ignorance or non-compliance with security measures, which can threaten the security of personal data, can be politically incorrect, offensive or even discriminatory, therefore the personal responsibility of AI users is very crucial. Guidelines for the use of AI have been developed at the national level and in educational institutions, facilitating its use in education.

The most important recommendations for educators and education policymakers are based on the results of this and other studies – “focus should be on proper teacher training, thoughtful integration of AI tools, and robust review processes for AI-generated content” (Matos et al., 2025).

## Data Availability

The data analyzed in this study is subject to the following licenses/restrictions: no. Requests to access these datasets should be directed to TP, tamara.pigozne@lu.lv.
